# Personal Support Networks of Older Adults in Relation to Their Perceptions About Disaster Management

**DOI:** 10.1093/geroni/igaa057.1420

**Published:** 2020-12-16

**Authors:** Sato Ashida, Lena Thompson, Emily Hejna, Sefonobong Obot, Haley Schneider

**Affiliations:** 1 University of Iowa College of Public Health, Iowa City, Iowa, United States; 2 University of Iowa College of Public Health, Iowa City, United States

## Abstract

Personal support networks are essential in ensuring the health and well-being of older adults especially in handling disaster situations. This study assessed the characteristics of personal support networks of older adults in relation to their perceptions about disaster management. Adults between ages 63 and 88 from Eastern Iowa participated in a survey prior to receiving a disaster preparedness education program. About half (47%) were living alone, 66% were female, and 53% had a high school diploma or fewer years of education. Forty-seven participants identified 308 support network members, with an average network size of 6.55 members ranging from 2 to 17. A greater number of network members with whom participants discussed disaster/emergency plans was associated with higher perceived response efficacy (preparation/planning “will help handle the situation better”; r=0.45, p=0.002), lower perceived barriers (preparation is “difficult to do”; r=-0.34, p=0.021), and higher self-efficacy to handle disaster situations (r=0.32, p=0.030). Having more members who would help “if something went wrong” was associated with higher self-efficacy to prepare (r=0.44, p=0.002) and to handle disaster situations (r=0.34, p=0.021). Fatalistic perception that nothing can help handle disaster situations was associated with having fewer people who provide emotional support (r=-0.559, p=.003) and who participants trust (r=-0.46, p=.018). Older adults’ social support network members may partly determine their perceptions regarding the importance of and their ability to prepare for emergency and disaster situations. Future studies may evaluate whether activating network member support and discussion would lead to increased motivation and preparedness among older adults.

